# Thermostable Direct Hemolysin Downregulates Human Colon Carcinoma Cell Proliferation with the Involvement of E-Cadherin, and β-Catenin/Tcf-4 Signaling

**DOI:** 10.1371/journal.pone.0020098

**Published:** 2011-05-20

**Authors:** Pinki Chowdhury, Debasis Pore, Nibedita Mahata, Poulomee Karmakar, Amit Pal, Manoj K. Chakrabarti

**Affiliations:** Division of Pathophysiology, National Institute of Cholera and Enteric Diseases, Beliaghata, Kolkata, India; Yale Medical School, United States of America

## Abstract

**Background:**

Colon cancers are the frequent causes of cancer mortality worldwide. Recently bacterial toxins have received marked attention as promising approaches in the treatment of colon cancer. Thermostable direct hemolysin (TDH) secreted by *Vibrio parahaemolyticus* causes influx of extracellular calcium with the subsequent rise in intracellular calcium level in intestinal epithelial cells and it is known that calcium has antiproliferative activity against colon cancer.

**Key Results:**

In the present study it has been shown that TDH, a well-known traditional virulent factor inhibits proliferation of human colon carcinoma cells through the involvement of CaSR in its mechanism. TDH treatment does not induce DNA fragmentation, nor causes the release of lactate dehydrogenase. Therefore, apoptosis and cytotoxicity are not contributing to the TDH-mediated reduction of proliferation rate, and hence the reduction appears to be caused by decrease in cell proliferation. The elevation of E-cadherin, a cell adhesion molecule and suppression of β-catenin, a proto-oncogene have been observed in presence of CaSR agonists whereas reverse effect has been seen in presence of CaSR antagonist as well as si-RNA in TDH treated cells. TDH also triggers a significant reduction of Cyclin-D and cdk2, two important cell cycle regulatory proteins along with an up regulation of cell cycle inhibitory protein p27^Kip1^ in presence of CaSR agonists.

**Conclusion:**

Therefore TDH can downregulate colonic carcinoma cell proliferation and involves CaSR in its mechanism of action. The downregulation occurs mainly through the involvement of E-cadherin-β-catenin mediated pathway and the inhibition of cell cycle regulators as well as upregulation of cell cycle inhibitors.

## Introduction

Colorectal cancer is the second leading cause of cancer and cancer related mortality in the world [1]. It is the third most common form of cancer and is most prevalent in industrialized developed nations [1]. A number of reports suggest that the under developed countries which are more susceptible to diarrhoeal diseases, are less prone to colorectal cancer. An inverse relationship has been observed between colorectal cancer and enteric infections [2]. Currently several approaches have been made to use bacteria or their products in the treatment of cancer [3]–[5]. A significant suppression of subcutaneous tumours in mice has been observed by combining anaerobic bacteria with several chemotherapeutic agents [6]. More recently Azurin, a small globular metalloprotein of *Pseudomonas aeruginosa* has been found to be capable of inducing apoptosis in tumour cells by p53 stabilization, makes this protein suitable for being employed as an anticancer agent [7], [8]. Furthermore, Pitari *et al.* [9] have shown that *Escherichia coli* heat stable enterotoxin (STa) suppress proliferation of colon carcinoma cell (T84) by increasing intracellular c-GMP. It has also been reported that heat stable enterotoxin (STa) secreted by enterotoxigenic *E. coli* downregulates human colon carcinoma cell (COLO-205) proliferation via PKG-ERK44/42 mediated signaling [10]. In correlation with these therefore, in the present study an attempt has been made to evaluate the role of thermostable direct hemolysin (TDH), secreted by *V. parahaemolyticus* and involved in gastrointestinal disorders [11], in the regulation of colon carcinoma cell proliferation.

There are reports that TDH may induce diarrhoea by elevation of the intracellular calcium through activation of calcium influx in intestinal epithelial cells [12]–[14]. It is well known that calcium ion (Ca^2+^) is a universal secondary messenger and a key player in many cellular signal transduction pathways [15], [16]. Several studies have shown that Ca^2+^ plays a crucial role in prevention of colon carcinogenesis [17], [18]. Ca^2+^ opposes tumorigenesis by restricting proliferation through promotion of E-cadherin expression and inhibition of β-catenin/Tcf-4 signaling [19], [20]. It is also known that high extracellular calcium promotes differentiation and decreases the rate of cell proliferation in human intestinal epithelial cells [21]–[23]. As TDH causes an increase in intracellular calcium level in intestinal epithelial cells through activation of calcium influx from extracellular environment and calcium-sensing receptor plays a vital role in influx of extracellular calcium, hence in this study we evaluate the potential of TDH in the down-regulation of colonic carcinoma cell proliferation (COLO 205). Our study reveals that TDH is capable of inducing reduction of cell proliferation. It has been found that this effect could be nullified by using CaSR si-RNA, indicating the involvement of this molecule in the mechanism of action of the toxin. Moreover, to understand the molecular mechanism of down regulation of cell proliferation we have also analyzed the role of E-cadherin, β-catenin/Tcf-4, Cyclin-D and the cell cycle inhibitory protein p27^Kip1^.

## Materials and Methods

### Maintenance of cell culture and preparation of viable cells

COLO-205 and HT-29 cell lines were purchased from NCCS, Pune, India. The cells were routinely cultured in tissue culture flasks and grown upto monolayers in RPMI-1640 and DMEM medium (Gibco BRL, USA) respectively, supplemented with 10% Fetal Bovine Serum (Gibco BRL, USA). Human fetal colonic epithelial cell line (CRL-1831) procured from ATCC (Rockville, MD) was maintained in DMEM:F-12 supplemented with 10% fetal bovine serum (FBS), 25 mM HEPES (Sigma), 10 ng ml^−1^ cholera toxin (Sigma), 5 µg ml^−1^ insulin (Sigma), 5 µg ml^−1^ transferrin (Sigma) and 100 ng ml^−1^ hydrocortisone (Sigma). The confluent monolayers were subcultured at the interval of 3–4 days. The viability of the cells was routinely checked by trypan blue (Gibco BRL, USA) exclusion. About 90±5% cells were remained viable.

### TDH purification

TDH was purified to homogeneity from *Vibrio parahaemolyticus* (strain number I-12366, serotype O3:K6) as described previously [24].

### si-RNA transfection

The expression of CaSR was blocked by transfection with si-RNA (Santa Cruz Biotechnology, USA) by using the manufacturer's protocol. The COLO-205 cells were transiently transfected with CaSR si RNA for 24 h. The transfected cells were then used for subsequent assays. For negative control experiments, scrambled si-RNA (Santa-Cruz Biotechnology, USA) was used.

### RNA isolation, reverse transcriptase-polymerase chain reaction and sequencing of calcium sensing receptor (CaSR)

Total RNA was isolated from transfected and non-transfected COLO-205 cells by using the manufacturer's protocol (Ambion, USA) and the concentrations were checked spectrophotometrically (Bio-Rad, USA). Total RNA sample (2 µg) was reverse transcribed into cDNA using Retro Script kit (Ambion, USA). The complementary DNA served as template for the amplification of CaSR and GAPDH by PCR in an automated thermal cycler (Eppendorf). The primer sequences for CaSR (forward: 5′-AATCTCG AGATGGCATTTTATAGCTGC-3′; reverse: 5′-GCGGTACCTTATGAATTCACTAC GTTTT-3′), and GAPDH (forward: 5′-GAAGGTGAAGGTCGGAGTC-3′; reverse: 5′-GAAGATGGTGATGGGATTTC-3′) were used for amplification. In case of negative control no RT product was used. The purified PCR product was subjected to sequence analysis by following the modified dideoxy chain termination method (Applied Biosystems model 3100 genetic analyzer automated sequencer).

### Immunocytochemistry

The presence of CaSR in transfected and non-transfected COLO-205 cells were observed by immunocytochemical method. Briefly, the COLO-205 cells were grown separately on lysine coated glass coverslips for 24 h. After incubation, the coverslips were washed with Hank's balanced salt solution (calcium free) followed by fixation using 4% paraformaldehyde in PBS for 20 min. After washing, the cells were permeabilized with 0.2% triton X-100 in PBS for 10–15 min with the subsequent incubation in 1% BSA prepared in PBS for 1 h. The cells were then washed and incubated with primary monoclonal antibody of CaSR (1∶100 dilution; ABR Affinity Bioreagents, USA) for 2 h followed by the incubation with FITC-conjugated secondary antibody (1∶1000 dilution; Jackson Immunochemicals, USA) for 1 h in dark. Finally the cells were washed thoroughly and observed under confocal microscope (Zeiss LSM 510).

### Measurement of free Ca^2+^ level

COLO-205 (l0^6^ cells ml^−1^) cells were loaded with Fura-2AM (5 µM) (Sigma, USA), a cell permeable calcium ion binding dye in the dark at 37°C for 30 min in PBS-glucose (pH 7.2) (Gibco BRL, USA) [25], [26]. The COLO-205 cells loaded with Fura-2AM were allowed to incubate in the absence (containing either 200 nM or 1 mM CaCl_2_) or presence of 10 µg ml^−1^ of TDH [27] at 37°C for different time point in PBS-glucose containing 200 nM CaCl_2_, 1 mM CaCl_2_ (Sigma, USA), 1 mM GdCl_3_ (Sigma, USA), a potent CaSR agonist with 200 nM CaCl_2_, 40 nM CaSR si-RNA (Santa Cruz Biotechnology, USA) with 200 nM CaCl_2_ and 1 mM EGTA (Sigma, USA), an extracellular calcium chelator for the each separate experiment. After washing with PBS the cells were analyzed with excitation at 340 nm and emission at 510 nm by FACS-Calibur using CELLQUEST software (Becton Dickinson).

### Cell proliferation assay

The extent of cell proliferation in presence of TDH was determined by CyQuant® NF cell proliferation assay kit (Molecular Probes, USA) by following the manufacturer's protocol. This is a non-radioactive assay based on the measurement of cellular DNA content via fluorescent dye binding [28]. COLO-205 cells were seeded into 96-well opaque-walled plates at a density of 1×10^4^ per well (100 µl) and they were allowed to adhere for 6 h at 37°C. Then the cells were treated with either media (containing either 200 nM or 1 mM CaCl_2_), protease digested TDH in presence of 200 nM CaCl_2_ or TDH in presence of 200 nM CaCl_2_, 1 mM CaCl_2_, 1 mM GdCl_3_ with 200 nM CaCl_2_, 40 nM CaSR si-RNA with 200 nM CaCl_2_ and 10 µM NPS-2390 with 200 nM CaCl_2_ (in separate sets) for 12 h. The cell growth medium was then removed and 100 µl of 1× lysis/dye binding solution was added into each well. The plate was incubated at 37°C for 15 min, and the fluorescence intensity was measured on a Microplate Reader (Bio-Rad) with a wavelength of 485 nm for excitation and 530 nm for emission. According to the calibration curve the fluorescence intensity is directly proportional to the cell growth. Each assay was done in triplicate and the mean results have been expressed as percent cell growth.

### LDH-cytotoxicity assay

Lactate dehydrogenase activity was measured by using the LDH Cytotoxicity Detection kit (Roche), according to the manufacturer's instruction. COLO-205 cells were seeded at 5×10^5^ cells in 96-well plates. Cells were then incubated in the absence or presence of different concentrations of TDH for 12 h. After treatment, the cell free supernatants were collected and then used in the LDH assay.

### Apoptosis assay

Apoptosis was measured by the TUNEL (TdT-dUTP terminal nick-end labelling) assay. Cells were plated in the 2-well glass slides (Nunc). After 48 h of incubation with TDH (10 µg/ml), the ApopTag® peroxidase kit (Millipore, USA) was used to detect in situ apoptosis according to the manufacturer's protocol. Briefly, cells (approximately 5×10^6^ cells/ml) were fixed in paraformaldehyde, and post-fixed in ice-cold ethanol/acetic acid (2∶1 v/v). After applying the equilibration buffer, the TdT (terminal deoxy-nucleotidyl transferase) was added, followed by anti-dioxigenin conjugate. Slides were stained with peroxidase substrate, counterstained by methyl green, dehydrated by xylene, and mounted under a glass coverslip to be viewed under the microscope at 400 magnification.

### Western blot analysis

COLO-205 cells were incubated without (in presence of either 200 nM or 1 mM CaCl_2_) or with 10 µg ml^−1^ of TDH for 1 h in presence of 200 nM CaCl_2_, 1 mM CaCl_2_, 1 mM GdCl_3_ with 200 nM CaCl_2_, 40 nM CaSR si-RNA with 200 nM CaCl_2_ (in separate sets). Treated and untreated COLO-205 whole cells were washed with ice cold phosphate buffered saline containing 1 mM Na_3_VO_4_, lysed in 50 µl of lysis buffer [20 mM Tris– HCl, pH- 8, 137 mM NaCl, 10% glycerol (v/v), 1% Triton X-100 (v/v), 1 mM Na_3_VO_4_, 2 mM EDTA, 1 mM PMSF, 20 µM leupeptin and 0.15 units/ml aprotonin] for 20 min at 4°C. The lysates were centrifuged at 15,000 g for 15 min and the supernatants (containing Triton X-100 soluble proteins) were collected. Then they were subjected to 10% SDS-PAGE and transferred to nitrocellulose membrane and immunoblotted with monoclonal antibodies of CaSR, β-Catenin, E-Cadherin, Kip1p27 and Cdk2 (Santa Cruz Biotechnology, USA), then incubated with alkaline phosphatase conjugated secondary antibody (Jackson Immunochemicals, USA). Actin was used as an internal control and detected with anti-β-actin monoclonal antibody (Santa Cruz Biotechnology, USA).

### Densitometric analysis

Immuno reactive bands of each blot were photographed and then images were digitized and analyzed by using Bio-Rad QUANTITY 1 software of the gel documentation system. The immunoreactive bands were quantitated and expressed as the ratio of each band density to the internal control (β-actin) band density.

### Statistical Analysis

The statistical significance was analyzed by Student's t-test (two-tailed) using SPSS 7.5 software. The results were expressed as the mean ± standard error of the mean (S.E.M.) where applicable, of three independent experiments. Statistical significance was assumed at p<0.01.

## Results

### Thermostable Direct Hemolysin (TDH)

**Figure 1:**
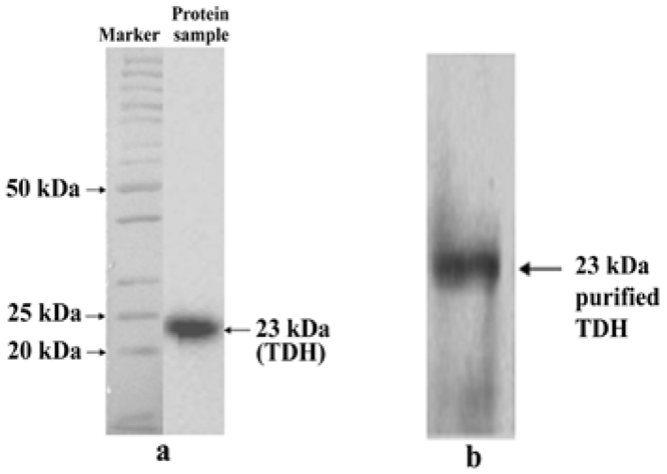
SDS-PAGE and western blot analysis of Thermostable Direct Hemolysin (TDH). (a) Sodium dodecyl sulfate-polyacrylamide gel electrophoresis showing the purification of the TDH of *Vibrio parahaemolyticus*. Lane1- Protein molecular weight marker (Fermentus). Lane2- Purified TDH (23 kDa). (b) Western blot analysis of purified 23 kDa TDH with anti-TDH antibody, produced by immunization of mice with purified TDH of *Vibrio parahaemolyticus*.

After purification the protein was subjected to a 10% SDS-PAGE and a single band of molecular weight 23 kDa was obtained (Figure 1a), suggesting that the molecular weight of the monomeric TDH is 23 kDa. The subsequent western blot analysis also confirmed the 23 kDa size of purified TDH (Figure 1b).

### Expression of CaSR mRNA and presence of CaSR protein in COLO- 205 cells

**Figure 2:**
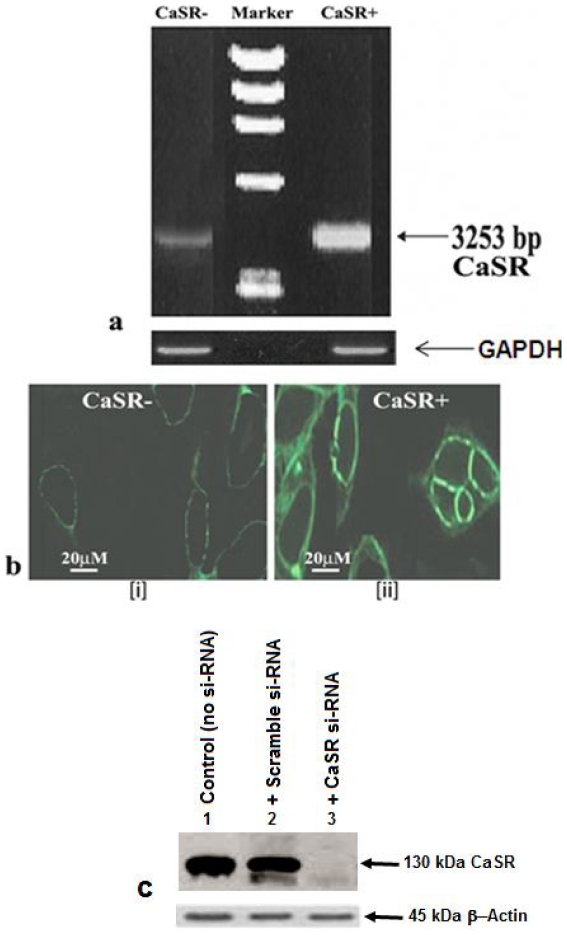
Expression of CaSR mRNA and presence of CaSR protein in COLO- 205 cells. (a) RT-PCR product of amplified calcium sensing receptor (CaSR) cDNA. Lane1- Amplified product of CaSR cDNA (3253 bp) in si-RNA transfected COLO-205 cells. Lane2- DNA molecular weight marker (Bangalore Genei). Lane3- Amplified product of CaSR cDNA (3253 bp) in non-transfected COLO-205 cells. (b) Immunocytochemical study showing the predominance of CaSR protein at the plasma membrane in [i] si-RNA transfected COLO-205 cells and [ii] non-transfected COLO-205 cells. The data shown are representative of three independent experiments. (c) Immunoblot analysis of the expression of CaSR in COLO-205. COLO-205 cell lysates were separated by SDS-PAGE, transferred to the membrane and finally probed with monoclonal antibody to CaSR. Lane 1, cells without CaSR si-RNA; Lane 2, cells in presence of scramble si-RNA (as negative control); and Lane 3, cells with CaSR si-RNA. β-Actin was used as loading control. The data shown are representative of three independent experiments.

In order to confirm the expression of CaSR mRNA, RT-PCR was performed on transfected and non-transfected COLO-205 cells. Our data demonstrated that the expression of full-length cDNA of CaSR (3253 bp) was noticeably reduced in transfected cells compared to that of non-transfected one but the expression levels of GAPDH gene remained same in both (Figure 2a). The identity of the PCR product was confirmed by nucleotide sequencing using an automated sequencer, which showed 96% homology with respect to that of the published sequence (NCBI Accession No. D50855) (data not shown). The CaSR protein expression was further confirmed by immunocytochemistry and immunoblotting. The immunocytochemistry analysis showed the major localization of the protein at the plasma membrane in non-transfected cells (Figure 2b [ii]). In transfected cells very less expression was found (Figure 2b [i]). To further confirm the presence of CaSR in COLO-205 cells, immunoblotting was performed and it was observed that both the non-transfected and scrambled siRNA transfected cells illustrated the expression CaSR, while the expression of CaSR was found to be completely abolished in CaSR siRNA transfected cells (Figure 2C).

### Change of cytosolic free Ca^2+^ level

**Figure 3:**
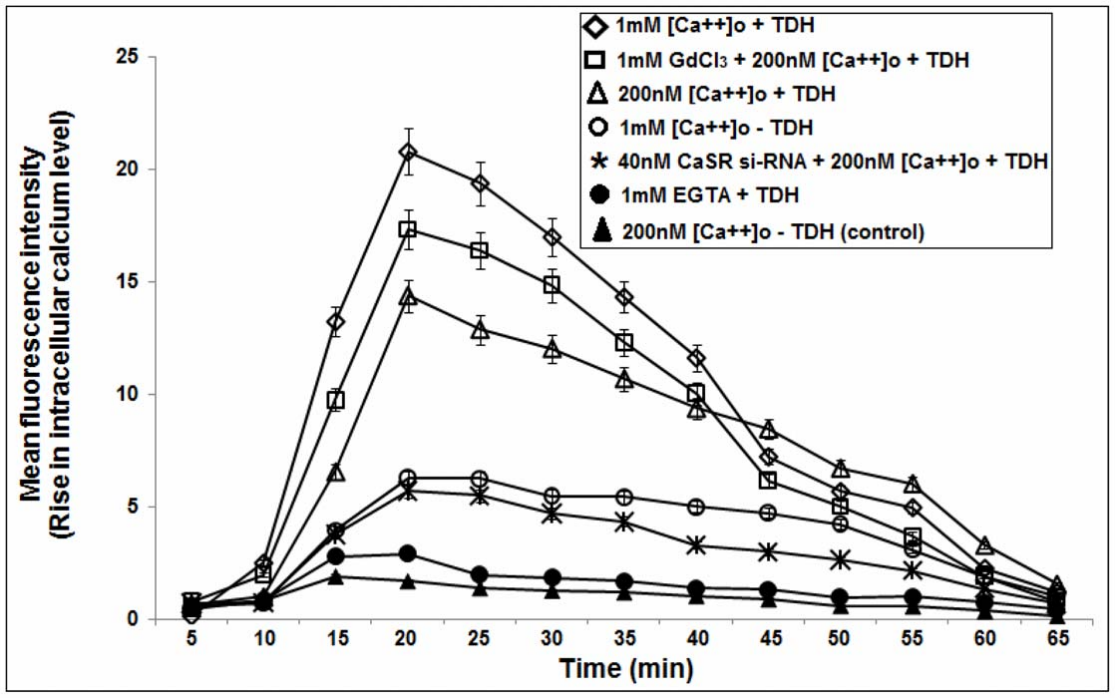
TDH induces cytosolic free Ca^2+^ level. Time dependent study of cytosolic free Ca^2+^ rise in COLO-205 cells after treatment with 10 µg/ml TDH. MFI, mean fluorescence intensities (arbitrary units) are measured by flow cytometry in the control (untreated) cells where 200 nM (▴) and 1 mM [Ca^2+^]_o_ (○) is present and in TDH treated cells in presence of 200 nM [Ca^2+^]_o_ (Δ); 1 mM [Ca^2+^]_o_ (◊); 1 mM GdCl_3_ and 200 nM [Ca^2+^]_o_ (□); 40 nM CaSR si-RNA and 200 nM [Ca^2+^]_o_ (*); 1 mM EGTA (•). Data are obtained from four independent experiments.

Experiments using different doses of purified TDH on the rise of [Ca^2+^]_i_ revealed that the maximum effect was achieved with 10 µg ml^−1^ of TDH concentration (data not shown). So, the subsequent studies were done by using this concentration. Time-dependent assay of intracellular calcium rise was done with purified TDH in COLO-205 cells using Fura-2AM, a cell permeable calcium ion binding fluorescence dye. It was observed that TDH causes a time-dependent rise of intracellular calcium [Ca^2+^]_i_ in the presence of 1 mM extracellular calcium [Ca^2+^]_o_ and the maximum effect was found after 20 min of incubation compared to control (MFI value 1.89 vs. 20.78, p<0.01, n = 3) (Figure 3). Chelation of [Ca^2+^]_o_ by EGTA inhibited the rise of [Ca^2+^]_i_ by TDH from MFI value 14.36 to 2.89 (p<0.01, n = 3) (Figure 3) suggesting that TDH-mediated [Ca^2+^]_i_ rise is due to the influx of calcium ion from extracellular environment. To evaluate the involvement of calcium sensing receptor in TDH-mediated calcium influx, we used 1 mM GdCl_3_, a potent CaSR agonist [29] in the extracellular medium in presence of 200 nM [Ca^2+^]_o_ and observed the rise of [Ca^2+^]_i_ which also reached to its maximum level at 20 min of incubation with TDH with respect to control (MFI value 1.89 vs. 17.33, p<0.01, n = 3) (Figure 3). It was observed that the TDH-mediated increase of [Ca^2+^]_i_ levels can be prevented by transfecting COLO-205 cells with CaSR si-RNA (MFI value 5.68 vs. 14.36, p<0.01, n = 3) (Figure 3) whereas in the negative control experiment the effect of scrambled si-RNA was not observed (data not shown). These data indicated that CaSR may be involved in the mechanism of intracellular calcium rise by TDH.

### Effect of TDH and CaSR in the regulation of cell proliferation

**Figure 4:**
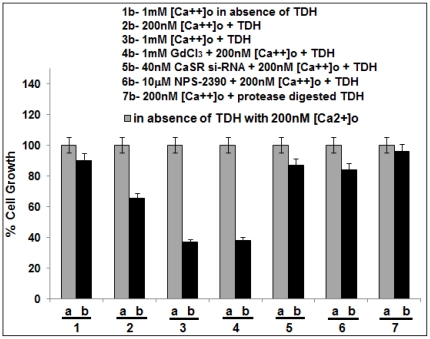
Effect of CaSR agonists and antagonists on TDH (10 µg/ml) induced downregulation of COLO-205 cell proliferation with respect to control. TDH untreated cells (control) has been considered as 100% cell growth. Data represent the mean ± SEM (*n* = 3). The bars 1a, 2a, 3a, 4a, 5a, 6a and 7a represent the untreated (control) COLO-205 cells in each case in presence of 200 nM [Ca^2+^]_o_ (100% cell growth). The others bars represent the TDH treated COLO-205 cells as follows, Bar 1b- TDH untreated in presence of 1 mM [Ca^2+^]_o_ (91.01% cell growth) Bar 2b- TDH treated in presence of 200 nM [Ca^2+^]_o_ (64.28% cell growth). Bar 3b- TDH treated in the presence of 1 mM [Ca^2+^]_o_ (36.67% cell growth). Bar 4b- TDH treated in the presence of 1 mM GdCl_3_ and 200 nM [Ca^2+^]_o_ (38.24% cell growth). Bar 5b- TDH treated in the presence of 40 nM CaSR si-RNA and 200 nM [Ca^2+^]_o_ (87.05% cell growth). Bar 6b- TDH treated in the presence of 10 µM NPS-2390 and 200 nM [Ca^2+^]_o_ (83.29% cell growth). Bar 7b- Protease digested TDH with 200 nM [Ca^2+^]_o_(95.78% cell growth) The data represent mean ± S.E.M. of three independent experiments, p<0.05.

To observe the involvement of CaSR in TDH mediated downregulation of cell proliferation, the COLO-205 cells were incubated with TDH in presence of 200 nM [Ca^2+^]_o_ and 64.28% of cell growth was observed compared to that of untreated one, contains only 200 nm [Ca^2+^]_o_ (which was considered as 100% of cell growth) (Figure 4). Treatment with TDH in presence of 1 mM [Ca^2+^]_o_ and GdCl_3_ in separate sets showed 36.67% and 38.24% of cell growth respectively (Figure 4). However, incubation with 10 µM NPS-2390, a potent CaSR antagonist (Sigma, USA) [19] in TDH-treated cells caused 83.29% cell growth. Transfection with CaSR si-RNA decreased the cell growth to 87.05% (Figure 4) whereas scrambled si-RNA reverted the phenomenon (data not shown). These results suggested that TDH causes downregulation of COLO-205 cell proliferation and CaSR may have a potent role in this mechanism of action.

### Lactase dehydrogenase and TUNEL assay

**Figure 5:**
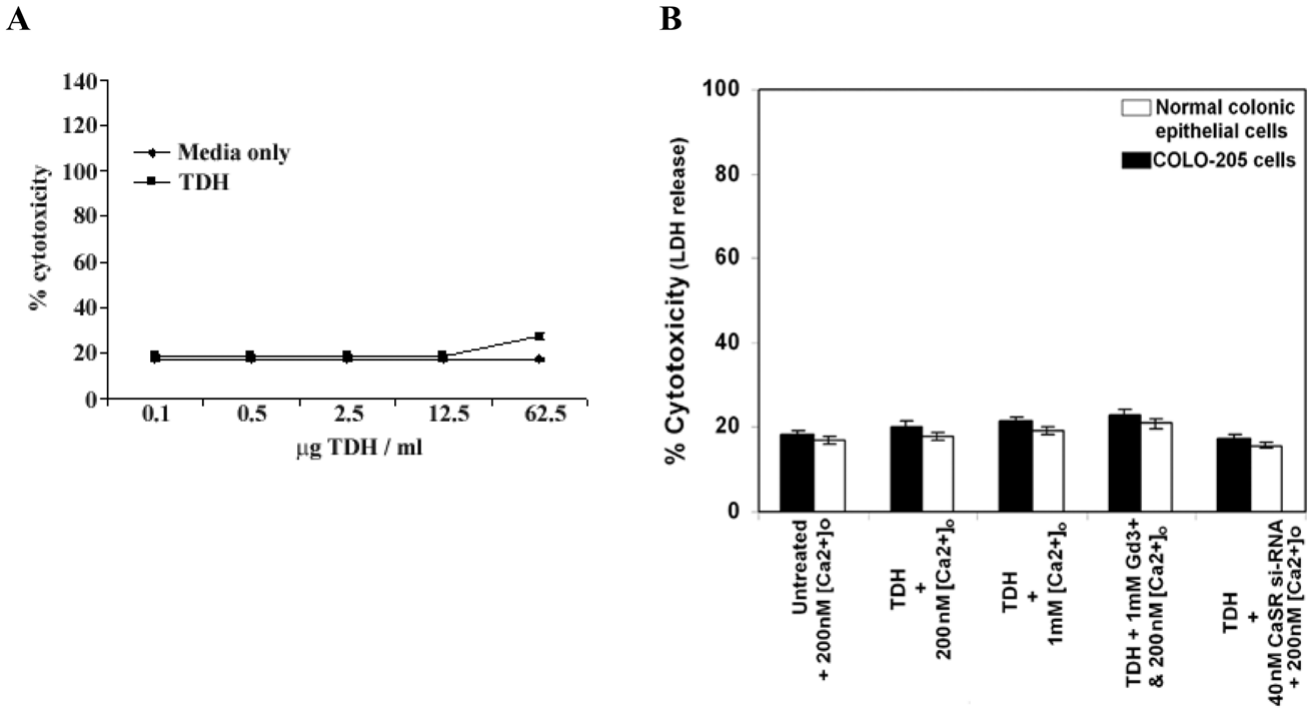
Lactate dehydrogenase (LDH) assay. A: Lactate dehydrogenase (LDH) release into the medium from COLO-205 cells incubated in presence TDH. COLO-205 cells were incubated without or with different concentration of TDH for 12 h. Cell free supernatants were collected and assayed. Data represent mean ± S.E.M. of three independent experiments, p<0.05. B: Percent cytotoxicity (LDH release) measurements in colonic carcinoma (COLO-205) cells (▪) and normal human fetal colonic epithelial cells (□). Data represent the mean ± SEM (*n* = 3), p<0.05.

**Figure 6:**
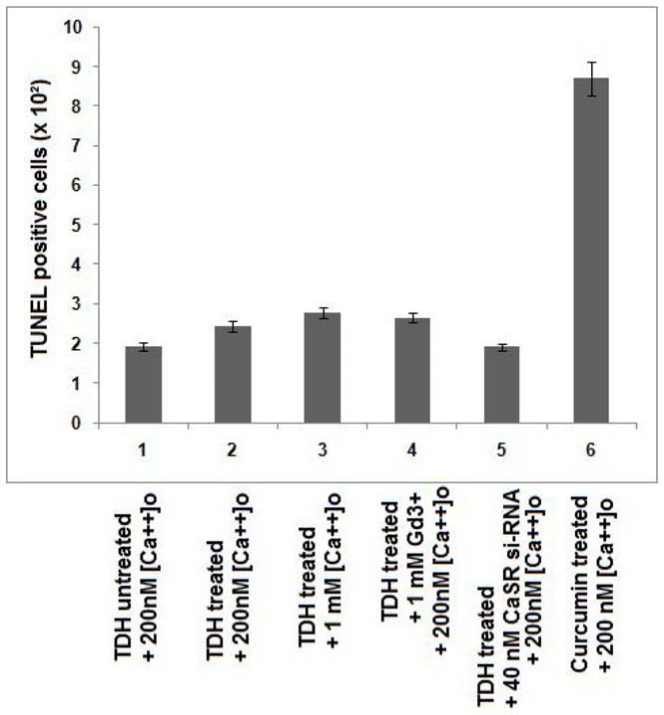
Quantitation of the TUNEL assay in TDH treated COLO-205 cells. The data are the mean values of five digitized images from three independent experiments.

To determine whether the downregulation of cell proliferation by TDH is due to cytotoxicity or apoptosis, lactase dehydrogenase (LDH) and TUNEL assay were performed. At first we performed a dose response study of TDH on COLO-205 cells with respect to LDH release. We did not find cytotoxic activity of TDH till the concentration of 12.5 µg/ml compared to untreated control. A little cytotoxicity was observed at a very high concentration of the toxin (62.5 µg/ml) (Figure 5a). Our results also revealed that the CaSR agonists ([Ca^2+^]_o_ and GdCl_3_) and inhibitor (si-RNA) had no significant effect on the release of LDH in TDH treated (10 µg ml^−1^ for 12 h) COLO-205 cells and normal human fetal colonic epithelial cells (Figure 5b). Moreover, it was examined that TDH did not induce cell death as confirmed by TUNEL staining (Figure 6).

### Effect of TDH on expressions of E-cadherin and β-catenin levels in COLO-205 cells

**Figure 7:**
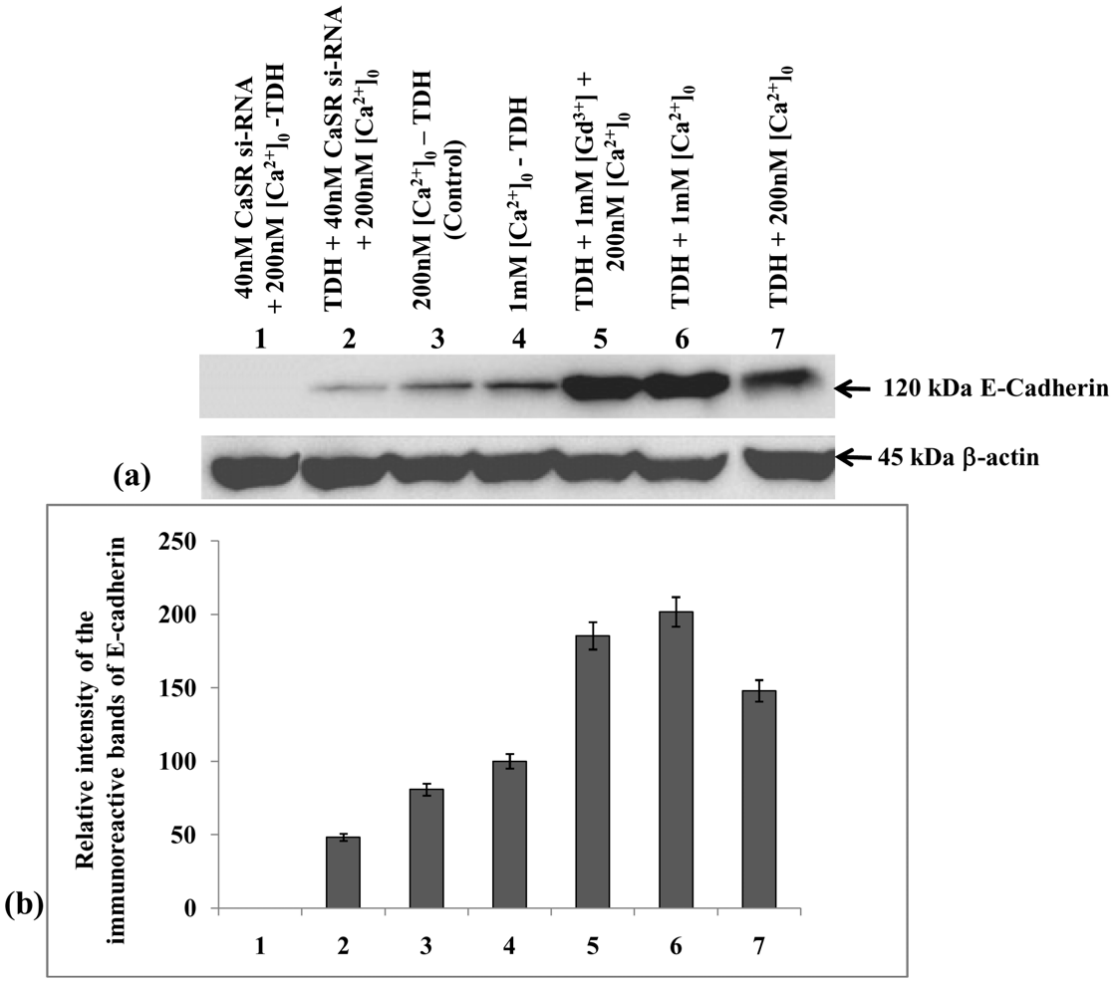
Role of TDH on E-cadherin expression in presence of CaSR agonist and antagonist in COLO-205 cells by Western Blot analysis. (a) Representative western blot analysis of E-cadherin expression. Data represent mean ± S.E.M. of three independent experiments, p<0.05. (b) Densitometric analysis of immunoreactive bands of E-cadherin of COLO-205 cell. After western blot, immunoreactive bands are photographed and then images are digitized and analyzed. Immunoreactive bands are quantitated and expressed as the ratio of each band density to the internal control (β-actin) band density. Bar 1- TDH untreated in presence of 200 nM [Ca^2+^]_o_ and 40 nM CaSR siRNA. Bar 2- TDH treated in presence of 200 nM [Ca^2+^]_o_ and 40 nM CaSR siRNA. Bar 3- TDH untreated in presence of 200 nM [Ca^2+^]_o_. Bar 4- TDH untreated in the presence of 1 mM [Ca^2+^]_o_. Bar 5- TDH treated in the presence of 1 mM GdCl_3_ and 200 nM [Ca^2+^]_o_. Bar 6- TDH treated in presence of 1 mM [Ca^2+^]_o_. Bar 7- TDH treated in the presence of 200 nM [Ca^2+^]_o_.

**Figure 8:**
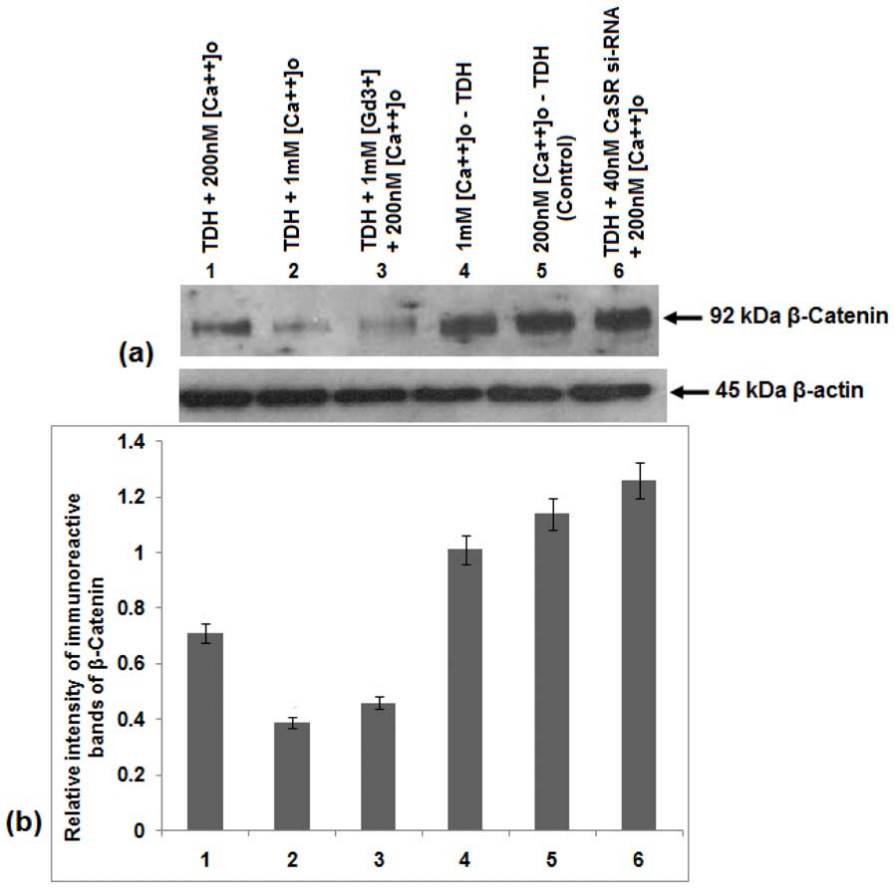
Role of TDH on β-catenin expression in presence of CaSR agonist and antagonist in COLO-205 cells by immunoblot analysis. (a) Representative western blot analysis of β-catenin expression. Data represent mean ± S.E.M. of three independent experiments, p<0.05. (b) Densitometric analysis of immunoreactive bands of β-catenin of COLO-205 cell. After western blot, immunoreactive bands are photographed and then images are digitized and analyzed. Immunoreactive bands are quantitated and expressed as the ratio of each band density to the internal control (β-actin) band density. Bar 1- TDH treated in presence of 200 nM [Ca^2+^]_o_. Bar 2- TDH treated in the presence of 1 mM [Ca^2+^]_o_. Bar 3- TDH treated in the presence of 1 mM GdCl_3_ and 200 nM [Ca^2+^]_o_. Bar 4- TDH untreated in the presence of 1 mM [Ca^2+^]_o_. Bar 5- TDH untreated in the presence of 200 nM [Ca^2+^]_o_. Bar 6- TDH treated in the presence of 40 nM CaSR si-RNA and 200 nM [Ca^2+^]_o_.

It has been reported earlier that E-cadherin (a well known adhesion molecule) and β-catenin (a proto-oncogene) have potent regulatory roles in cell proliferation [19]. To evaluate the involvement of E-cadherin and β-catenin in the TDH mediated downregulation mechanism of COLO-205 cell proliferation, western blot analysis of E-cadherin and β-catenin were done. Promotion of E-cadherin level occurred when the cells were incubated with TDH in presence of 1 mM [Ca^2+^]_o_ and GdCl_3_ in separate sets but the level decreased in presence of CaSR si-RNA compared to that of the control (untreated) (Figure 7). Completely reverse phenomenon was observed in case of β-catenin. Figure 8 showed that suppression of β-catenin level occurred in TDH-treated cells as well as both in presence of 1 mM [Ca^2+^]_o_ and GdCl_3_, but CaSR si-RNA reverted the effect of TDH in inhibition of β-catenin expression level. These results suggest that TDH downregulates cell proliferation through the CaSR-mediated pathway by the upregulation of E-cadherin and reduction in β-catenin levels.

### Effect of TDH on the expressions of Cyclin D, cdk2 and p27^Kip1^ levels in COLO-205 cells

**Figure 9:**
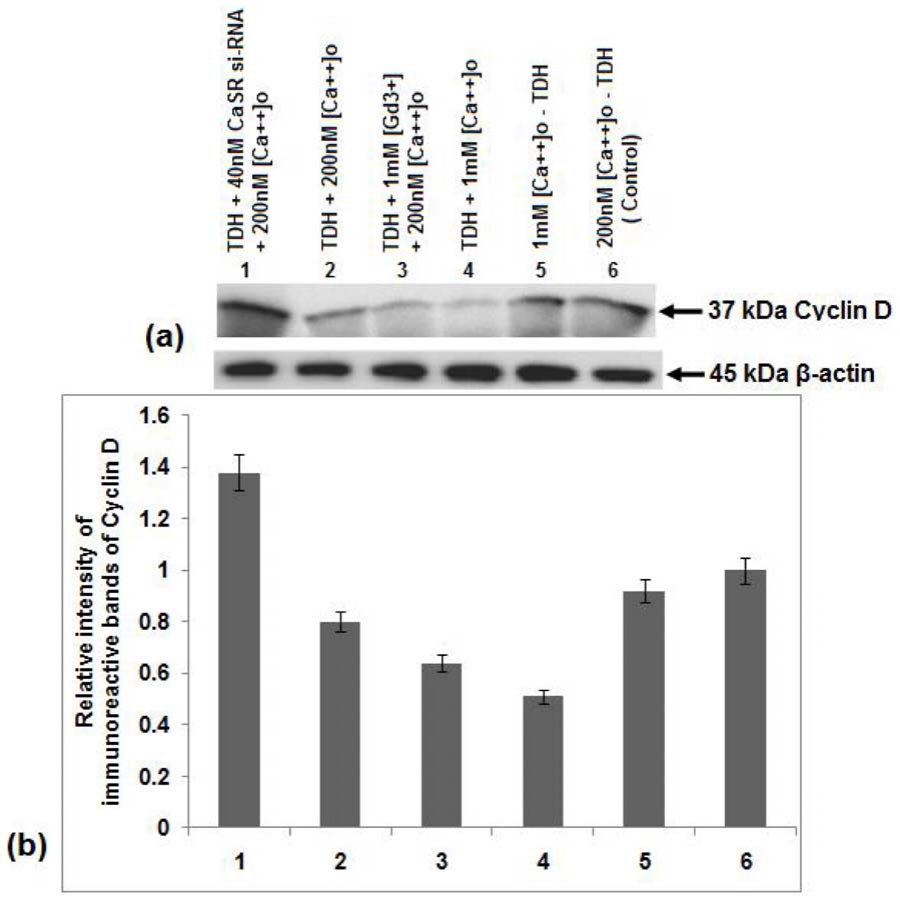
Role of TDH on Cyclin D expression in presence of CaSR agonist and antagonist in COLO-205 cells by immunoblot analysis. (a) Representative western blot analysis of Cyclin D expression. Data represent the mean ± SEM (*n* = 3). (b) Densitometric analysis of immunoreactive bands of Cyclin D of COLO-205 cell. After western blot, immunoreactive bands are photographed and then images are digitized and analyzed. Immunoreactive bands are quantitated and expressed as the ratio of each band density to the internal control (β-actin) band density. Bar 1- TDH treated in the presence of 40 nM CaSR si-RNA and 200 nM [Ca^2+^]_o_. Bar 2- TDH treated in presence of 200 nM [Ca^2+^]_o_. Bar 3- TDH treated in the presence of 1 mM GdCl_3_ and 200 nM [Ca^2+^]_o_. Bar 4- TDH treated in the presence of 1 mM [Ca^2+^]_o_. Bar 5- TDH untreated in the presence of 1 mM [Ca^2+^]_o_. Bar 6- TDH untreated in the presence of 200 nM [Ca^2+^]_o_.

**Figure 10:**
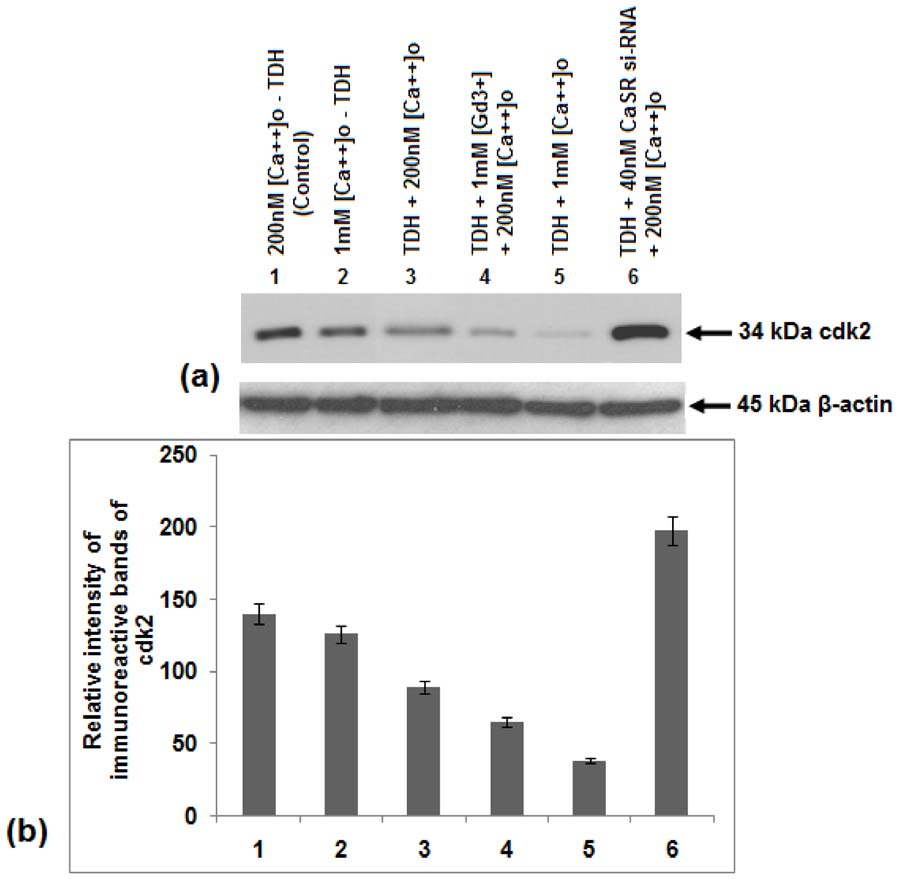
Role of TDH on cdk2 expression in presence of CaSR agonist and antagonist in COLO-205 cells by Immunoblotting. (a) Representative western blot analysis of cdk2expression Data represent the mean ± SEM (*n* = 3). (b) Densitometric analysis of immunoreactive bands of cdk2 of COLO-205 cell. After western blot, immunoreactive bands are photographed and then images are digitized and analyzed. Immunoreactive bands are quantitated and expressed as the ratio of each band density to the internal control (β-actin) band density. Bar 1- TDH untreated in presence of 200 nM [Ca^2+^]_o_. Bar 2- TDH untreated in presence of 1 mM [Ca^2+^]_o_. Bar 3-TDH treated in the presence of 200 nM [Ca^2+^]_o_. Bar 4- TDH treated in the presence of 1 mM GdCl_3_ and 200 µM [Ca^2+^]_o_. Bar 5- TDH treated in presence of 1 mM [Ca^2+^]_o_. Bar 6- TDH treated in the presence of 40 nM CaSR si-RNA and 200 nM [Ca^2+^]_o_.

**Figure 11:**
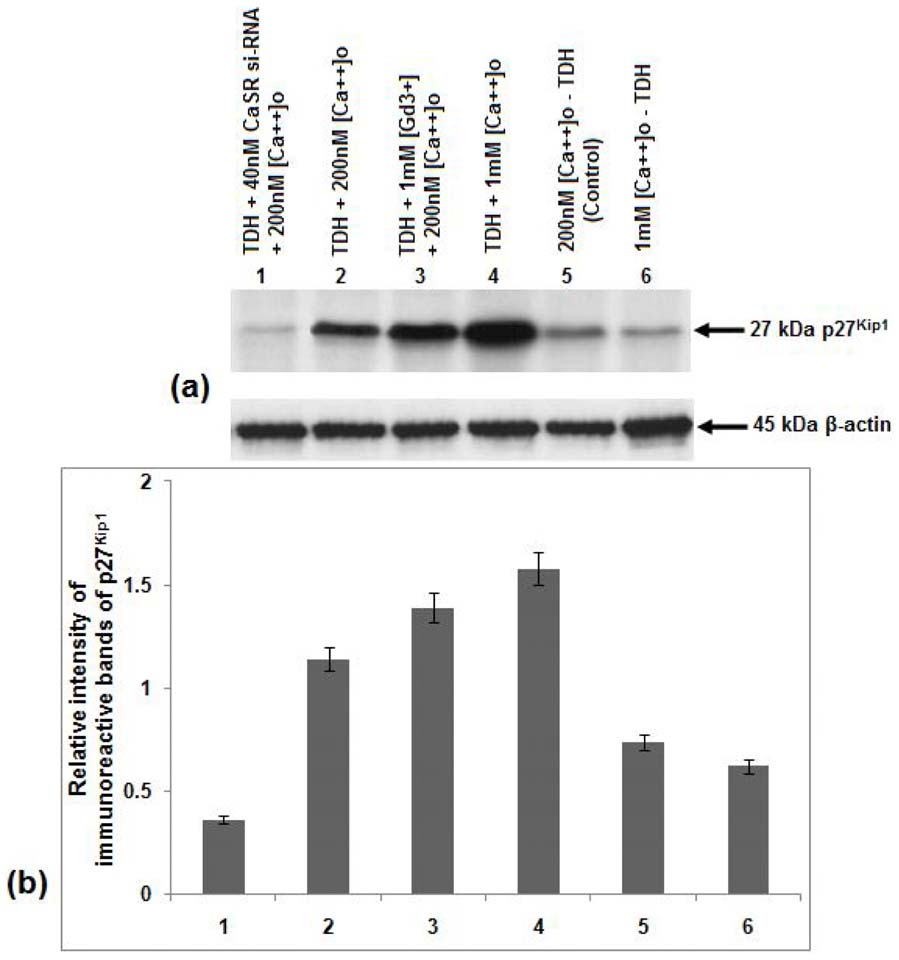
Role of TDH on p27^Kip1^ expression in presence of CaSR agonist and antagonist in COLO-205 cells by immunoblot analysis. (a) Representative western blot analysis of p27^Kip1^expression Data represent the mean ± SEM (*n* = 3). (b) Densitometric analysis of immunoreactive bands of p27^Kip1^ of COLO-205 cell. After western blot, immunoreactive bands are photographed and then images are digitized and analyzed. Immunoreactive bands are quantitated and expressed as the ratio of each band density to the internal control (β-actin) band density. Bar 1- TDH treated in the presence of 40 nM CaSR si-RNA and 200 nM [Ca^2+^]_o_. Bar 2- TDH treated in presence of 200 nM [Ca^2+^]_o_. Bar 3- TDH treated in the presence of 1 mM GdCl_3_ and 200 nM [Ca^2+^]_o_. Bar 4- TDH treated in the presence of 1 mM [Ca^2+^]_o_. Bar 5- TDH untreated in the presence of 200 nM [Ca^2+^]_o_. Bar 6- TDH untreated in the presence of 1 mM [Ca^2+^]_o_.

Cyclin D and cdk2 are two important regulatory molecules that are associated with the G1-S phase transition of cell cycle and expression of these two molecules are significantly correlated with the degree of malignancy in a variety of human carcinomas [30]–[33]. In this report, activity of Cyclin D and cdk2 were shown by western blot analysis. It was observed that TDH treatment inhibited Cyclin D level in COLO-205 cells. The degree of inhibition increased further when the cells were treated with TDH along with 1 mM [Ca^2+^]_o_ and GdCl_3_ but prior treatment with CaSR si-RNA increased the Cyclin D level in TDH-treated cells (Figure 9). Similar result was noticed in case of cdk2 expression level also (Figure 10). It is well known that the cdk inhibitors p21^Cip1^ and p27^Kip1^ play important roles in mediating growth arrest and are thought to function as brakes of the cell cycle [34]. In the present study, immunoblot analysis with anti- p27^Kip1^ antibody showed that the CaSR agonists [Ca^2+^]_o_ or GdCl_3_ raised the p27^Kip1^ level but inhibition of its expression occurred in TDH treated CaSR si-RNA transfected cells (Figure 11), suggesting that TDH causes downregulation of COLO-205 cell proliferation and involve CaSR through the reduction of cell cycle regulatory proteins e.g. Cyclin D, cdk2 and activation of cdk inhibitor e.g. p27^Kip1^ level.

## Discussion

The ability of many live or attenuated pathogenic bacteria or their components to cause cancer regression has been well known for many years [3], [4]. The most prominent example would be the use of *Mycobacterium bovis* BCG, the vaccine strain, in the treatment of bladder cancer [35]. The ability of *Salmonella*, *Clostridia*, and other anaerobic bacteria to target tumors for their preferential replication, leading to tumors regression has been reported by Dang et al [36]. Dang et al. used the chemotherapeutic agent mitomycin C and the antivascular agent dolastatin-10 in combination with the spores of an attenuated anaerobic bacterium, *Clostridium novyi*, to treat colorectal cancer cells [36]. A couple of macrolides, epothilone A and epothilone B (EpoA and EpoB) from the myxobacterium *Sorangium cellulosum* and particularly a chemically modified synthetic form of EpoB, desoxy epothilone B, have been shown to have antitumor activity against a range of human tumors [37]. More recently, purified redox proteins such as azurin have been shown to allow cancer regression in nude mice harboring human melanoma [7]. It has also been observed that bacterial enterotoxin suppress colon carcinoma cell proliferation based on calcium dependent pathway [9], [10]. These studies inspired us to use thermostable direct hemolysin (TDH), a well-known traditional virulence factor of *V. parahaemolyticus*, in the regulation of colon carcinoma cell proliferation (COLO-205). *Vibrio parahaemolyticus*, a marine bacterium secretes thermostable direct hemolysin (TDH), which is associated with diarrhoea [12]. It has been reported by Takahashi *et al.*, 2000 [27] that TDH involves calcium ions in its mechanism of action and 10 µg ml^−1^ causes influx of [Ca^2+^]_o_ with the subsequent rise of [Ca^2+^]_i_ in human intestine. It is also known that the influx of [Ca^2+^]_o_ occurs through different receptors present on the plasma membrane e.g. calcium sensing receptor (CaSR). CaSR is activated not only by [Ca^2+^]_o_ but also by several polyvalent cations e.g. GdCl_3_ and raises [Ca^2+^]_i_ level [29]. Moreover, extracellular calcium promotes differentiation and decreases growth in the intestinal epithelial cells [38]–[40]. A report suggests that activation of CaSR might be an important event in the control of cell proliferation [41]. Therefore, in the present work we have determined the role of CaSR in the mechanism of action of TDH mediated downregulation of COLO-205 cell proliferation.

Our study reveals that purified TDH raises intracellular calcium concentration in presence of extracellular calcium and maximum effect has been noticed after 20 min of incubation of cells in presence of TDH. Chelation of extracellular calcium by EGTA inhibits the rise in intracellular calcium level by TDH, which corroborates the findings of Fabbri *et al.*, 1999 [12] that TDH raises intracellular calcium level through calcium influx from extracellular environment. The rise of [Ca^2+^]_i_ in TDH-treated cells has also been observed in presence of GdCl_3_, a potent CaSR agonist [19]. However, no significant intracellular calcium rise has been observed after transfecting COLO-205 cells with CaSR si-RNA, suggesting that CaSR becomes activated both in presence of calcium and gadolinium in TDH-treated COLO-205 cells and increases [Ca^2+^]_i_. Moreover, it has been found that TDH inhibits cell proliferation in presence of extracellular calcium and gadolinium but CaSR si-RNA and NPS-2390 revert the inhibitory effect of TDH with respect to TDH untreated (control) cells which has been considered as 100% cell growth. Incomplete recovery of cell growth to the control level in presence of CaSR inhibitors suggests the possible existence of an alternative TDH-mediated signaling pathway other than involvement of CaSR.

The reduction of cell proliferation rate can be explained either by cell death or reduced proliferation. TUNEL assay and LDH-release assay have been done to investigate whether treatment of TDH causes apoptosis and cytotoxicity in COLO-205 cells. Moreover, we are also interested to observe whether TDH has any cytotoxic effect in normal colonic epithelial cells. A very few TUNEL positive COLO-205 cells have been observed after TDH treatment, whereas a significantly high number of positive cells have been seen after treatment with curcumin as an apoptotic inducer in positive control experiment. Furthermore, no significant cytotoxicity has been found at a low concentration of TDH. Significant cytotoxicity has been observed at a very high concentration of the protein. These results correlate with the previous finding of Hiyoshi et al., 2010 [42] and Raimondi et al., 2000 [14], where cytotoxicity of TDH has been shown at a high dose. Although the study of Raimondi et al. [14], demonstrates that the high toxin dose exhibits calcium-independent cytotoxicity in Caco-2 cells. Our present study reveals that the effect of TDH is extracellular calcium dependent in COLO-205 cells. Moreover, there is no statistically significant difference in LDH-release between the control and TDH treated COLO-205 cells as well as normal colonic epithelial cells both in presence of CaSR agonists and inhibitor. The same experiments have been repeated in case of HT-29, another colon carcinoma cell line and the results obtained are similar like that of COLO-205 (data not shown). In contrast to the observation made by Naim *et al.*, 2001 [43] in rat-1 cells, our results suggest that apoptosis and cytotoxicity are not contributing to the reduction of proliferation rate in colon carcinoma cells and this reduction appears to be caused by decrease in cell proliferation.

It has been reported earlier that E-cadherin and β-catenin have potent regulatory role in colonic cell proliferation [19]. E-cadherin plays a key role as a tumor suppressor in colon carcinoma cells, and up-regulated expression of E-cadherin correlates with the induction of differentiation [44]. We examined the expression of E-cadherin by immunoblotting analysis. Our results reveal that TDH in presence of 1 mM Ca^2+^ and 1 mM Gd^3+^ induces the expression of E-cadherin, but the expression of E-cadherin has noticeably been reduced in CaSR si-RNA transfected cells following TDH treatment. It is well known that E-cadherin interacts with β-catenin, a component of the TCF/Wnt signaling pathway, and activation of Wnt is involved with malignancy [45]. To evaluate the involvement of β-catenin on COLO-205 cell proliferation, western blot analysis of β-catenin has been done. The results demonstrate that TDH suppresses β-catenin level in presence of [Ca^2+^]_o_ and GdCl_3_, whereas a reverse effect has been observed in CaSR si-RNA transfected cells. This suggests the involvement of CaSR in the downregulation of cell proliferation by TDH.

Moreover, the expressions of Cyclin D and cdk2, two important molecules associated with the G1-S phase transition of cell cycle are also inhibited by TDH in [Ca^2+^]_o_ as well as GdCl_3_ treated cells. Their expression levels are significantly correlated with the degree of malignancy in a variety of human carcinomas [30]–[33]. It is well known that the cdk inhibitors e.g. p27^Kip1^ play an important role in mediating growth arrest and are thought to function as brakes of the cell cycle [34]. In the present study immunoblotting with anti- p27^Kip1^ antibody suggests that TDH raises the cdk inhibitory proteins and helps to pause the cell cycle progression in presence of CaSR agonists but the level of p27^Kip1^ decreases when CaSR is inhibited by its si-RNA. These results indicate the possible involvement of CaSR in TDH mediated downregulation of cell proliferation.

In conclusion, we have stated for the first time that thermostable direct hemolysin can downregulate cell proliferation in COLO-205 cells and involves calcium sensing receptor in its mechanism of action. The downregulation occurs mainly through the involvement of E-cadherin-β-catenin mediated pathway and the inhibition of the cell cycle regulatory proteins e.g. Cyclin D, cdk2 as well as upregulation of cdk inhibitor e.g. p27^Kip1^ level. This study clearly helps in developing our understanding that TDH may be considered as a novel agent in the therapy of human colorectal cancer in future.
